# Supplementary material for: Molecular and Metagenomic Analyses Reveal High Prevalence and Complexity of Viral Infections in French-American Hybrids and North American Grapes
Source: Viruses. 2023 Sep 19;15(9):1949. doi: 10.3390/v15091949 (PMC10534776; doi:10.3390/v15091949)
Supplement: Supplementary file 1 [file viruses-15-01949-s001.zip › viruses-2531532-supplementary.pdf]

viruses

MDPI

Supplemental materials

Table S1. Primers used in RT-PCR (or PCR) detection of target viruses. For the full names of viruses, refer to the list of abbreviations.

Viruses	Primers	Sequences	Amplicon (bp)	Target gene
GRBV	GRBV685F	GAGGGTTGTTTGAAGATAAAG	719	CP
	GRBV1403R	CCATAATAAACAGCGTGGTC		
GLRaV-1	LR1-502F	TTGAGRGCTCTBATAAAYGAAC	379	HSP70h
	LR1-880R	CGTTMARTTCGYCKACSGACA		
GLRaV-2	LR2-14568F	RCDATGGAGYTRATGTCYGA	525	CP
	LR2-15092R	AGCGTACATRCTYGCRAACA		
GLRaV-3	LR3-CP107F	TCTTAAARTAYGTTAAGGACGG	301	CP
	LR3-CP407R	GGCTCGTTAATAACTTTCGG		
GLRaV-4	LR4-13269F	GGACAATTTAGGTAATGTWGTRGCTAC	490	P23 (ORF6)
	LR4-13758R	TATCCTCAGWGAGGAARCGG		
GLRaV-7	LR7-12163F	CTAGTGAATTACACCGAGAAGTC	550	CP
	LR7-12712R	GTGACTTGGCACGCATGTATC		
GRSPaV	RSP35	AGRYTTAGRGTRGCTAARGC	476	Rep
	RSP36	CACATRTCATCVCCYGCAAA		
GPGV	GPGV6586F	GAYATGTCGATTCGTCAGGAG	436	CP
	GPGV7021R	CGACTTCTGGTGCCTTATCAC		
GVA	GVA6538F	TCTTCGGGTACATCGCCTTG	325	CP
	GVA6862R	TCRAACATAACCTGTGGYTC		
GVB	GVB6448F	ATGGAAAATATATCCCKGATGG	603	CP
	GVB7050R	GTTAACCACCTATATYTCRACAG		
GFkV	GFkV5161F	TGGAAAGCYTGCCAGACCCT	377	Rep
	GFkV5537R	TCGTCRTABGTDCCTGGTTC		
ToRSV	ToRSV7330F	TTCTGGGATTCTAATCGTACG	302	3′UTR
	ToRSV7613R	TGCAACTGCACGAGTATTATC		
GFLV	GFLV3135F	TTGAGATTGGWTCYCGTTTC	558	CP
	GFLV3692R	CTGTCGCCACTAAAAGCATG		
ArMV	ArMV2291F	CRGGTATTACGTGGGTTATGAG	292	CP
	ArMV2582R	GCTGCCTCAAACTCAGCATA		

CP: capsid protein; HSP70h: heat shock protein 70 homologue; Rep: replicase protein; UTR: untranslated region.

Table S2. Summary of sequence contigs from Baco samples and their mapping to viruses and viroids. For the full names of viruses, refer to the list of abbreviations.

Viruses	GenBank accession no.	Reference isolates	Sequence identity (%)	No. of contigs	Genome coverage (%)
GRSPaV	KX274274.1	SK704-A	98	1	99.8
GPGV	KR528587.1	Tannat-GvPGV	99	1	100
GRBV	KY316023.1	ONRB5	99	1	100
GVB	GU733707.1	H1	96-97	3	45.5

Table S3. Summary of sequence contigs from Concord samples and their mapping to viruses and viroids. For the full names of viruses, refer to the list of abbreviations.

Viruses	GenBank accession numbers	Reference isolates	Sequence identity (%)	No. of contigs	Genome coverage (%)
GLRaV-3	GU983863.1	WA-MR	98-100	8	94.4
GRBV	KY426922	93-26	100	1	100
GRSPaV	AY368590.1	Syrah	96	1	52.6
	AY368590.1	Syrah	93-94	3	70.6
	KR054734.1	JF	98	6	99.2
	FR691076.1	MG	98	1	96.9
	KX274275.1	SK704-B	95	1	33.8
	HE591388	PG	89	1	65.7
	KX035000.1	SWTi clone 2	97	3	37.0
	JX559646.1	3138-07	86	1	31.1
GVB	KY426923	8415	84-88	4	30.1

Table S4. Summary of sequence contigs from Coronation and their mapping to viruses and viroids. For the full names of viruses, refer to the list of abbreviations.

Viruses	GenBank accession numbers	Reference isolates	Sequence identity (%)	No. of contigs	Genome coverage (%)
GRSPaV	KX035002.1	SGM4 Clone 1	92-97	6	60.2
GPGV	KU194413.1	BC-1	98-99	8	98
TSV	FJ403375.1	Illinois RNA1	99	2	73.1
	FJ403376.1	Illinois RNA2	99-100	2	69.6
	FJ403377.1	Illinois RNA3	91	1	91.7

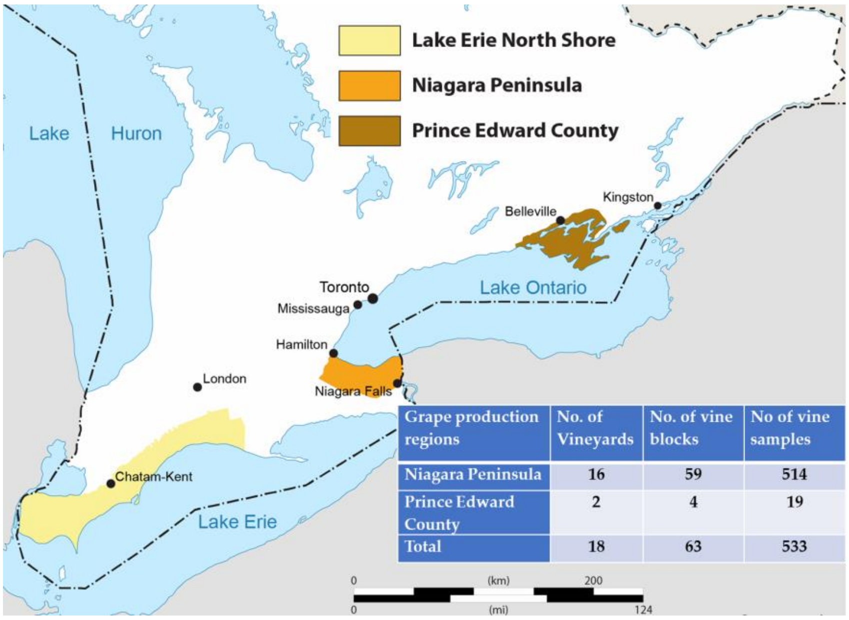

Grape production regions	No. of Vineyards	No. of vine blocks	No of vine samples
Niagara Peninsula	16	59	514
Prince Edward County	2	4	19
Total	18	63	533

Figure S1. Sampling of grapevines across two primary appellations in Ontario for use in virus survey. The map is from https://vineyards.com/wine-map/canada/ontario. The insert table shows the numbers of samples, numbers of vine blocks and vineyards that were sampled from the two appellations in the province.
